# Investigation of the Effect of Curcumin on Metabolic Dysfunction Caused by Clozapine in Rats

**DOI:** 10.1192/j.eurpsy.2024.709

**Published:** 2024-08-27

**Authors:** M. Aydin, U. Egilmez, D. Eryavuz, A. Unlu

**Affiliations:** ^1^Psychiatry; ^2^Biochemistry, Selcuk University, Konya, Türkiye

## Abstract

**Introduction:**

Antipsychotics disrupt intracellular cholesterol traffic and prevent the exit of low-density lipoprotein (LDL)-derived cholesterol from the endosome/lysosome compartment. It was showed that curcumin accelerated the release of cholesterol-containing exosomes from cells with impaired intracellular cholesterol traffic due to antipsychotic treatment and suggested that curcumin may help minimize the negative metabolic effects associated with chronic antipsychotic treatment.

**Objectives:**

This study aimed to investigate the effectiveness of orally administered curcumin to rats in preventing and treating metabolic syndrome-related side effects such as weight gain and dyslipidemia caused by clozapine.

**Methods:**

In our research, a total of 32 male rats (Wistar Albino), 12 weeks old, produced at Selçuk University Experimental Research and Application Center, were used. All animals divided into 4 groups. Venous blood collection and weight measurements were taken from all groups at the beginning. 32 rats were randomly divided into 4 separate groups: control, only oral clozapine, oral clozapine + 50 mg/kg curcumin, and the oral clozapine + 100 mg/kg curcumin group. Groups II-III-IV were given 15 mg/kg clozapine orally daily for 3 weeks. AST, ALT, glucose, total cholesterol, Triglyceride, HDL, LDL and insulin were studied from the blood samples taken at the beginning and at the end of the experiment.

**Results:**

There was no statistically significant difference in comparisons of weight and insulin measurements between the groups at the end of the experiment (p>0.05). In glucose measurements at the end of the experiment, the control group was found to have significantly higher glucose values compared to the other groups (p <0.001). As a result of posthoc analyses, LDL measurements in the control group were found to be lower than those in the CLZ and CLZ +50 c groups (p<0.05). AST value of the control group was significantly higher than the CLZ+100c group (p=0.011). Measurements of the control group for ALT were found to be higher than those of the CLZ+50c and CLZ+100c groups (p<0.05). There was no statistically significant difference between the groups in HDL, TG, Cholesterol comparisons (p>0.05).

In the control group, the average weight at the end of the experiment was significantly higher than at the beginning (p = 0.045). In the CLZ group, the mean glucose at the end of the experiment was significantly lower than at the beginning (p<0.001).

**Conclusions:**

Metabolic problems due to antipsychotics negatively affect treatment compliance. Treatment support methods that can solve or help this problem may be useful. Our study ended in conflicting results. Needs for new experiments…

**Disclosure of Interest:**

None Declared

